# Evaluation of a Binary Classification Approach to Detect Herbage Scarcity Based on Behavioral Responses of Grazing Dairy Cows

**DOI:** 10.3390/s22030968

**Published:** 2022-01-26

**Authors:** Leonie Hart, Uta Dickhoefer, Esther Paulenz, Christina Umstaetter

**Affiliations:** 1 Competitiveness and System Evaluation, Agroscope, Tänikon 1, 8356 Ettenhausen, Switzerland; christina.umstaetter@thuenen.de; 2 Institute of Agricultural Sciences in the Tropics, University of Hohenheim, Fruwirthstrasse 31, 70599 Stuttgart, Germany; 3 Institute of Animal Nutrition and Physiology, Christian-Albrechts-Universität zu Kiel, Hermann-Rodewald-Strasse 9, 24118 Kiel, Germany; dickhoefer@aninut.uni-kiel.de; 4 Division of Animal Husbandry and Ethology, Faculty of Life Sciences, Humboldt University of Berlin, Invalidenstrasse 42, 10115 Berlin, Germany; esther.paulenz@hu-berlin.de; 5 Thuenen Institute of Agricultural Technology, Bundesallee 47, 38116 Braunschweig, Germany

**Keywords:** precision grazing management, herbage allowance, chewing behavior, rumen fill scoring, milk yield drop, allocating new pasture, decision support

## Abstract

In precision grazing, pasture allocation decisions are made continuously to ensure demand-based feed allowance and efficient grassland utilization. The aim of this study was to evaluate existing prediction models that determine feed scarcity based on changes in dairy cow behavior. During a practice-oriented experiment, two groups of 10 cows each grazed separate paddocks in half-days in six six-day grazing cycles. The allocated grazing areas provided 20% less feed than the total dry matter requirement of the animals for each entire grazing cycle. All cows were equipped with noseband sensors and pedometers to record their head, jaw, and leg activity. Eight behavioral variables were used to classify herbage sufficiency or scarcity using a generalized linear model and a random forest model. Both predictions were compared to two individual-animal and day-specific reference indicators for feed scarcity: reduced milk yields and rumen fill scores that undercut normal variation. The predictive performance of the models was low. The two behavioral variables “daily rumination chews” and “bite frequency” were confirmed as suitable predictors, the latter being particularly sensitive when new feed allocation is present in the grazing set-up within 24 h. Important aspects were identified to be considered if the modeling approach is to be followed up.

## 1. Introduction

Current sensor technology in farming offers decision support in addition to the farmer’s knowledge and experience. Binary systems indicate need or no need for action, thereby enabling farm staff to respond to crop and animal reactions in a timely manner.

In arable farming, many precision technologies have already been adopted; however, the implementation of such in the livestock sector is hugely important, as it can improve consumer trust and product traceability [1]. Sensor data gathered in livestock farming play a growing role for proactive, instead of reactive, management to improve animal production, health, and welfare [1,2,3]. The increasingly available data benefit animal identification and monitoring as well as the control of animal production according to current regulations [2]. Predictions on an individual animal basis that consider physiological evolvements over time and might even combine information of various sensors may have the advantage of detecting vulnerabilities in large herds before it is possible for humans, but they generate large amounts of data, which calls for machine learning approaches [4,5]. However, assessment of the generalizability of prediction models is often lagging behind and, thus, their commercial implementation and farmer’s adoption [1,6].

In livestock farming, cattle have been the most studied animal species for using the Internet of things and developing machine learning algorithms to improve management [4]. Non-invasive biometric sensor technologies that monitor behavior and physiological parameters in real time are an important information source for management decisions [1]. To date, many sensor technologies have focused on applications in indoor systems, because conditions are more steady, and technologies rely less on battery power than in pasture-based systems, but tools are increasingly being developed for use under grazing conditions as well [7].

Using behavioral sensor data combined with machine learning approaches is particularly of relevance in pasture-based dairy systems, because grazing animals are not under supervision at all times. For instance, a binary system could support the decision to allocate new pasture as soon as paddocks are grazed empty and feed intake of cows decreases. This could allow for more efficient herbage utilization in future, as farmers could respond to animal reactions with a pasture change before economic losses occur. Additionally, such an approach could be of great use in grazing areas that are difficult to reach under severe weather conditions.

With a known dry matter (DM) intake of cows, a precision grazing strategy can be pursued [8], and the timing of a pasture reallocation can be optimally chosen without wasting feed residues or using grasslands too intensively. However, measuring available herbage quantity by means of weekly farm walks using rising plate meters is laborious [9,10] and error-prone [11] and so are visual observations by humans. Measuring the available herbage quantity on pastures via unmanned aerial vehicles and satellites has been heavily investigated in recent years [12,13,14]. However, when coupled with grass growth models, this approach only allows for estimation of DM intake on a herd basis and not for individual animals. Moreover, it is difficult to monitor herbage DM intake of individual dairy cows, because it is influenced by multiple environmental and animal physiological factors [15] and, thus, highly variable.

Nevertheless, the decision of changing paddocks can also be based on changes in the animals’ behavior in response to declining herbage availability. A few studies have already ventured into this vision. For instance, Hamidi et al. [8] observed longer walking distances of suckler cows, and O’Driscoll et al. [16] found fewer and shorter lying bouts together with less time lying in dairy cows with low than with high herbage availability on pasture. Moreover, Werner et al. [17] observed, amongst other behavioral indicators, a reduced rumination activity and an increased grazing bite frequency under restricted, compared to unrestricted, herbage allowance in a one-day rotational system. This is consistent with the results of Rombach et al. [18], who included bite frequency during grazing as an important predictor, with a negative relationship in their estimation model of herbage DM intake.

The study by Werner et al. [17] was followed up by Shafiullah et al. [19], who used their data to train models via supervised learning that evaluated sufficiency or insufficiency of herbage allowance on a 24 h basis in grazing dairy cows. The idea was to support farmers in scheduling paddock rotations before cow performance dropped. Using a binary system, Shafiullah et al. [19] developed and validated several prediction approaches based on changes in the cows’ number of rumination chews per day and of their grazing bite frequency, along with six other variables. Among various statistical learning methods, a random forest model (RFM) performed best in classifying the binary feeding status of grazing dairy cows. The more classical generalized linear model (GLM) has performed slightly worse. The two approaches attained AUCs (area under receiver operating characteristic curve) of 88% (RFM) and 85% (GLM) using cross validation, which is satisfactory.

However, in order to correctly assess the generalizability of such models to different grazing conditions of dairy cows, and, thus, their possible implementation in future smart farming systems, they should be evaluated in commercial herds [6,20] and for a differing grazing system, such as a multi-day rotation. Variables selected and prediction models developed by Shafiullah et al. [19] have not yet been evaluated using independent data from another dairy farm.

The aim of the present study was, therefore, to evaluate two prediction models recommended by Shafiullah et al. [19] under commercial farming conditions. For this purpose, a practice-oriented rotational grazing system was set up with a gradually decreasing pasture allowance over six subsequent grazing days. Allocated paddocks yielded 20% less herbage DM than needed by the grazing cows. During the six-day grazing cycles, cow-individual days with feed scarcity were identified on the basis of the prediction models by means of behavioral changes and compared with reference indicators based on milk yield and rumen fill. More specifically, the two objectives were: (i) to investigate how well the model-predicted binary classification of “sufficient” and “scarce” pasture allowance for each cow and day agreed with the reference indicators; and (ii) to assess if the behavioral variables selected by Shafiullah et al. [19] to predict a pronounced feed insufficiency are also sensitive to a gradual decline in herbage availability.

## 2. Materials and Methods

In an experiment with commercial farming conditions, dairy cows grazed half-days in a rotational grazing set-up at the surrounding grasslands of a research barn during an entire grazing season. During three experimental months in May, July, and September, data were gathered. In these periods, the animals grazed for six days in an allowance-restricted pasture so that a herbage scarcity was achieved towards the end of the six-day grazing cycle. Animals were then allocated to a new paddock.

### 2.1. Grazing Site

**Figure 1:**
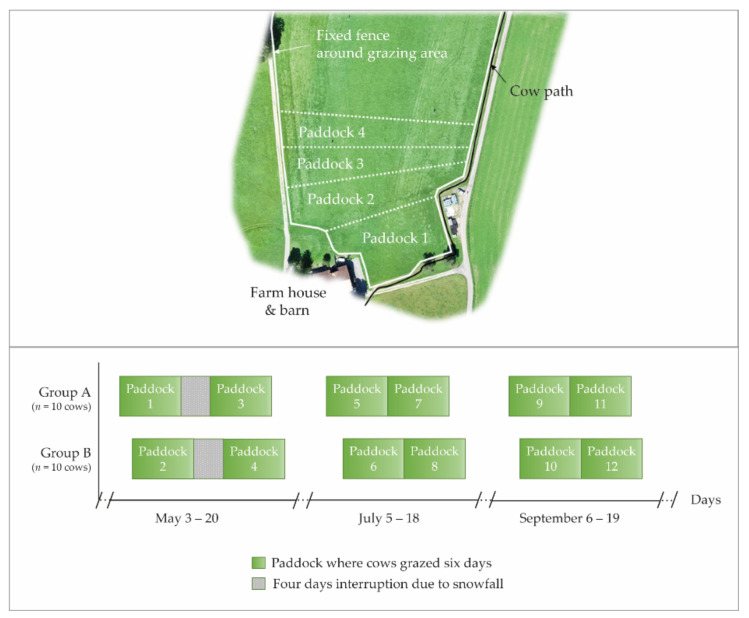
Schematic illustration of the grazing area indicating the spatial paddock arrangement for one experimental month (**top**) and the timeline of the allocation (**bottom**). The temporal allocation of paddocks for both Cow Groups A and B is shown as a rectangular box. Green shading indicates the gradually decreasing herbage allowance during the six-day grazing cycle.

The experiment was carried out at the Agroscope Dairy Research Barn Waldegg in 2019, located in the north-east of Switzerland (47°49′ N, 8°92′ E, and 534 m above sea level). While the cumulative annual precipitation was 1293 mm, average daily ambient air temperatures during the course of the year varied from −5.3 °C to 26.8 °C (agrometeo.ch, location: Wil, Saint Gall, Switzerland). A total grazing area of approximately 1.7 ha was used during the three experimental months (Figure 1). For the purpose of the experiment, this grazing area was divided into four adjacent paddocks per experimental month with varying sizes between 0.17 and 0.52 ha each, depending on available herbage DM per ha. The paddocks were grazed sequentially. At the beginning of each experimental month, the paddock rotation started again from the area closest to the barn. While the boundaries of the grazing area were fenced with a fixed electric fence, a mobile electric fence separated the paddocks. The soil beneath the grazing area was rich in clay (27%, determined by field texturing) and had a low humus content of 2.5–3.5%. The permanent grassland had not been grazed for several years but was used for silage and hay production. Approximately 80% of the area had been re-sown in 2015 with a grass mixture (28% perennial ryegrass, 72% other grasses) and the remaining area in 2013 with a grass-clover mixture (10% white and 4% red clover, 28% perennial ryegrass, 58% other grasses). Each paddock was spatially arranged to include both sward mixtures so that the botanical composition of their vegetation was very similar.

### 2.2. Animals and Housing

In total, 20 Brown Swiss cows were used, comprising seven primiparous and 13 multiparous cows in their second to fifth lactation, resulting in a mean lactation number of 2.4 (one standard deviation (SD): 1.2). At the beginning of the experiment, 19 cows were, on average, 142 days in milk (SD: 44) and one exceptional cow 420 days in milk. The average body weight of the cows at the beginning of the experiment was 626 kg (SD: 64) and their daily milk yield 29.1 kg (SD 6.3). Body weight of cows was determined using a common measuring tape for cattle that measured the circumference of the torso close to the front legs. Body condition scores (BCS) of cows were assessed by a well-trained dairy production advisor once before each experimental month using scores from 2 to 5, including 0.25 steps [21]. The cows had, on average, a BCS of 2.8 (SD: 0.4) in April before the experiment began and of 3.1 (SD: 0.5) in September, when it ended.

The cows were kept in two groups of 10 cows each, hereafter referred to as Group A and Group B. The groups represented a treatment repetition and differed only in that they were allocated to a new paddock with a temporal shift. Individual cows were assigned to the two groups so that the number of cows with and without grazing experience was balanced (i.e., always or never grazed before, respectively). In addition, the Cow Groups A and B were balanced with respect to average body weight (627 kg and 625 kg), milk yield (29.4 kg d^−1^ and 28.8 kg d^−1^), and BCS (2.9 and 2.8). The average lactation number slightly differed between the two groups (A: 2.7 and B: 2.0).

Cows were milked twice daily at 05:30 and 16:30 in a side-by-side milking parlor (Lemmer-Fullwood GmbH, Lohmar, Germany). Both groups were housed in separate areas of a cubicle free stall barn and grazed on separate paddocks. Throughout the entire vegetation period (mid-March to mid-November), cows grazed the permanent grassland for half-days in a multi-day rotational regime. Cows were kept on pasture between milkings from 07:00 to 16:00 during the spring and autumn, whereas, during the warm summer months (June to August), they grazed overnight from 18:00 to 05:00. During the remainder of the day, the animals were kept indoors, where they had access to roughages and concentrates offered at a feeding bank and a feeding station, respectively. Both on pasture and indoors, cows had free access to fresh drinking water. All experimental procedures were in accordance with the Swiss guidelines for animal protection and approved by the responsible Animal Experimental Committee under the file reference TG01/19.

### 2.3. Experimental Setup

Each group of 10 cows grazed on one paddock over six consecutive days (i.e., one grazing cycle; Figure 1). The allocated paddock area was calculated to supply 20% less than the group’s total DM requirement for maintenance and lactation for the entire six-day grazing cycle (see section below). As a consequence, the herbage allowance on pasture decreased from day 1 to day 6, when it was considered to have become scarce.

The six-day grazing cycles were repeated twice every experimental month, directly one after the other, with the exception of May, when grazing had to be interrupted for four days between both grazing cycles due to snowfall. To account for possible weather effects, the commencement of grazing cycles of the two groups was offset by two days (Figure 1).

Overall, a total of 720 individual observations were targeted (i.e., 3 experimental months × 2 grazing cycles × 6 days × 2 cow groups × 10 cows).

### 2.4. Feed Allocation

In order to adjust the paddock size and, thus, herbage allowance for the cows’ half-days on pasture and half-days in the barn, the daily DM requirements were estimated using an Excel-based calculation tool for feed advisors (Agridea FuPlan, version 7.90, Lindau, Switzerland). The tool took into account information on the nutritional quality of the supplement feeds and of the pasture vegetation from a feed database (www.feedbase.ch (accessed on 21 March 2019)) and the average body weight of the 20 cows as well as their average milk yield and concentrations of lactose, fat, and protein. The latter three parameters were measured by an accredited laboratory (ISO 9622 method; Suisselab AG, Zollikofen, Switzerland). The estimated daily herbage requirement was 11.3 kg DM per cow on pasture and 8.8 kg DM per cow of supplement feed offered in the barn, with 5.7 kg DM per cow of meadow hay as well as 3.1 kg DM per cow of dried whole-plant corn pellets in the barn (i.e., 100% feed allowance). From this, the feed allowance on pasture and in the barn was reduced by 20%. The restriction level of 20% was chosen to represent a practical on-farm scenario of a mild feed restriction, which modifies ingestive behavior [16,17,22] but does not dramatically reduce milk yield [23,24,25].

#### 2.4.1. Herbage Allowance

To determine the respective size of each paddock, the compressed sward height (CSH) was measured one day before each grazing cycle by using a rising-plate meter (RPM; Grasshopper^®^ G2 Sensor, App version 4.02, TrueNorth Technologies, Shannon, Ireland). Based on established calibrations per experimental month (see Section 2.5), average pre-grazing CSH (mm, *n* = 25) was converted into estimates for above-ground herbage mass (HM, kg DM ha^−1^), assuming herbage > 20 mm CSH is available for cows, because cows are able to graze rather deeply when they are at strongly restricted pasture allowance [17]. Daily herbage regrowth during the six-day grazing cycles of 90, 60, and 30 kg DM ha^−1^ were assumed for May, July, and September, respectively, according to the location’s altitude, soil type, climatic conditions, and time of the year [26]. Additionally, 10% of HM losses (on DM basis) were considered due to animal trampling and rejected patches.

Subsequently, the required size of the paddocks was calculated from the net available HM and the HM needed to achieve a herbage allowance on pasture of 80% of the animals’ requirements for herbage DM over the entire six-day grazing cycles. Then, paddocks were established using electric fences and the RPM’s global positioning module. Due to inaccuracies in measuring CSH and spatial coordinates, as well as to inaccuracies in estimating HM, the amount of allocated herbage varied between paddocks (Table 1).

#### 2.4.2. Allowance of Roughage and Concentrate Feeds Offered in the Barn

The amounts of both roughages (31 kg DM per group of dried whole-plant corn pellets and 57 kg DM per group of meadow hay) offered in the barn were also reduced by 20%. To ensure that all animals had the same access to roughages, cows were fixed one by one after they returned from milking. After milking and rumen fill scoring (see Section 2.6.2) were completed, roughages were made accessible to the whole group of cows simultaneously. For this, the roughages were distributed equally along the feeding banks of both groups once daily. Additionally, commercial dairy concentrates were fed to cows via a feeding station, with the amounts depending on their individual milk performance (average 0.7 kg DM cow^−1^ d^−1^; SD: 1.2). Daily amounts of both roughages and concentrate feeds offered were kept constant across each six-day grazing cycle, except meadow hay, which was offered ad libitum at day 1 of each grazing cycle to compensate possible effects of the noticeable feed restriction on the last day of a previous grazing cycle.

From day 2 on, refusals of the roughage feeds were weighed daily after cows spent the half-day in the barn. While cows refused on average 4.3 kg DM (SD 3.3; *n* = 12) per group of roughages across all grazing cycles and both cow groups on day 2 of each grazing cycle, daily amounts of residuals were very low during days 3 to 6 of each grazing cycle (average 1.1 kg DM per group; SD: 0.9; *n* = 12).

### 2.5. Pasture Measurements

Prior to each experimental month, i.e., in May, July, and September, a calibration was developed to estimate HM from CSH. For this, above-ground HM was harvested manually in 9–15 randomly selected but evenly distributed plots (0.5 m × 0.5 m) across the anticipated grazing area. Within the respective plots, an average CSH (*n* = 3) was determined beforehand. The fresh samples were weighed, dried in a ventilated oven at 60 °C for a minimum of 48 h, and weighed again to determine DM concentrations and the HM (kg DM ha^−1^). Subsequently, linear relationships between HM (y) and CSH (x) were established (Table A1).

After each daily grazing event, herbage availability on paddocks was monitored. To do so, the CSH was measured at 25 equally distributed points per paddock using the above-mentioned RPM to determine post-grazing CSH.

### 2.6. Animal-Related Measurements

#### 2.6.1. Behavior

To measure the lying and feeding behavior, all 20 cows were equipped with pedometers on the right foreleg (Lemmer-Fullwood GmbH, Lohmar, Germany) and halters with a noseband sensor (RumiWatch, Itin&Hoch, Liestal, Switzerland) at the beginning of each experimental month. Pedometers measured the daily number of lying bouts (i.e., LAYDOWN, Table 2) using a 3-axis accelerometer. They were automatically read when the animals entered the milking parlor. The noseband sensors measured jaw and head movements in a 10 Hz resolution using a pressure tube and pressure sensor on the cow’s nose in combination with a 3-axis accelerometer, which were both integrated into a halter around the head of the animal. The RumiWatch system allows for real-time transmission of measurements via the wireless networking protocol ANT+ and preliminary classification of behaviors so that users can check data plausibility. However, using the raw data to classify behaviors includes validity checks and makes the classification more accurate [27]. The raw data are saved on a memory card integrated in the halter and can be read via micro USB. User-defined post-processing of sensor data is then possible on a desktop computer using the RumiWatch Converter software. Technical specifications of the RumiWatch system and information about the algorithms used to classify the behaviors are given by Zehner et al. [27] and Werner et al. [17].

Eating, grazing, and rumination behaviors as well as head activity were extracted from the sensors’ raw data at a 1-hour resolution using the RumiWatch Converter V0.7.4.13. Grazing and rumination bouts were detected and their duration recorded, as described by Werner et al. [28].

The eight behavioral variables selected by Shafiullah et al. [19] were relevant for the purpose of the present study, i.e., the daily prediction of herbage scarcity (Table 2). Therefore, the hour-based behavioral variables—bite frequency, rumination chews per bolus, and head movement activity—were averaged across each day, whereas the daily sum was calculated for the following variables: daily rumination chews, daily rumination time, and eating bouts. Finally, the mean duration of a rumination bout (i.e., the variable “rumination time per bout”) was calculated by dividing the daily rumination time by the number of detected rumination bouts. Daily rumination times of < 117 min (*n* = 9), which was the minimum value in the dataset of Shafiullah et al. [19], were not considered plausible [29]. Therefore, nine observations were removed from the present dataset.

#### 2.6.2. Milk Yield and Rumen Fill

Milk yield of individual cows was automatically recorded by the milking system throughout the whole experiment and the daily sum of individual morning and evening milk yields computed (kg cow^−1^). Rumen fill was visually assessed from the depth of the paralumbar fossa as an indicator for the feed intake during the last four to six hours [30,31]. Rumen fill scoring was performed when cows were fixed at the feeding bank after the milking event that followed grazing and before roughages were offered. Therefore, it took place 1 to 1.5 h after cows had grazed last. Two well-trained direct observers assigned scores between 1 (low fill quantity) and 5 (high fill quantity), following the criteria of Zaaijer and Noordhuizen [30] and using the further developed protocol of Agroscope [32] that included 0.5 scores. The two observers showed high concordance in their scoring [33].

### 2.7. Reference Indicators for Herbage Scarcity

Both rumen fill scoring and milk yield served the purpose of having a practice-oriented reference indicator for evaluating the herbage availability experienced by individual cows. The first is relatively easy to determine by farmers themselves. Milk yield is usually automatically recorded and represents an important economic factor for the producer. The amount of milk a cow produces is strongly related to the cow’s DM intake [34,35,36].

In the present experiment, we deliberately chose not to use estimation models for DM intake during grazing as reference measurement. First, they are not validated yet to evaluate day-to-day differences in individual cows. Additionally, they are error-prone, depending on the measurability of their input variables and their variability under varying environmental conditions [18,37,38].

#### 2.7.1. Baseline Values

Cow individual baseline values for rumen fill score and milk yield were calculated by taking an average from days 1 and 2, in the case of rumen fill, and days 2 and 3 of each grazing cycle, in the case of milk yields. Therefore, changes in rumen fill score and daily milk yield relative to cows’ respective baseline values were used as indicators for scarce herbage availability and the subsequent low DM intake. For this, absolute changes in rumen fill scores and relative changes in milk yields were calculated for each cow for days 3 to 6 and 4 to 7, respectively. As daily milk yields gradually declined across the six-day grazing cycle in some cows until one day after each grazing cycle ended, in May and September we used the milk yield of the subsequent day to evaluate the herbage availability on the previous day (e.g., daily milk yield on day 4 as an indicator of the herbage availability on day 3). In July, when cows grazed overnight until 05:00, the daily sums of milk yields were used for that exact day and not the day after, because milk yields began to recover at the subsequent day already.

#### 2.7.2. Reference Classification

**Figure A1:**
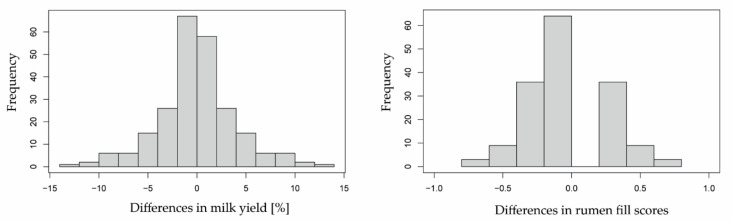
Histograms of differences in milk yield (**left**, *n* = 237) and rumen fill scores (**right**, *n* = 240) appearing during grazing days 1 and 2.

Declines in milk yield and rumen fill beyond the normal variation were considered to indicate whether herbage availability experienced by each cow on days 3 to 6 was either “sufficient” or “scarce”. To determine reduction limits of normal variation in either reference indicator, SDs in daily milk yields and rumen fill scores were calculated for each cow and each experimental month across days 1 and 2 and days 2 and 3, respectively, of both consecutive grazing cycles (*n* = 4). The average SD across all cows and experimental months in rumen fill scores was ± 0.38 (*n* = 60; Figure A1). Since milk yield of cows varied considerably, respective SDs were expressed relative to the mean per cow and experimental month, with an average of ± 5.9% (*n* = 57; three extreme outliers visually identified and excluded). These generated SD values were then multiplied by a factor of 3, which represents the 99.73% confidence interval of the standard normal distribution [39] (Figure A1), resulting in the reduction limits of 1.14 for rumen fill scores and 17.7% for milk yields. The reduction limits were interpreted as follows; compared to respective baseline values, absolute differences in rumen fill scores < 1.14 and relative differences in milk yields < 17.7% on days 3 to 6 were attributed to an expected normal day-to-day variation (class “no herbage scarcity” (=0)), whereas those above these limits were considered as a warning sign for scarce herbage availability (=1). For example, a reduction in milk yield by 20% compared to the baseline value was an indication of herbage scarcity. No herbage scarcity was assumed for days 1 and 2, used to calculate the baseline values (classification = 1).

### 2.8. Application of Prediction Models

The herbage availability was additionally predicted based on changes in the eight behavioral variables (Table 2) as determined by Shafiullah et al. [19]. The authors used, among others, GLM and RFM to predict the binary herbage availability status of one cow at one specific day, i.e., of a cow-day. The prediction models of Shafiullah et al. [19] were established and validated using a dataset from an Irish full-time grazing system with a 1-day rotation and two portions of new pasture strips per day [17]. The dataset included 1221 observations from 40 individual cows. Animal behavior was measured using the RumiWatch noseband sensors and pedometers as described above. In the Irish study, cows had been offered (in % of their DM intake capacity) 100% of herbage DM (“sufficient”, *n* = 629) or 60% of herbage DM (“insufficient”, *n* = 592) on pasture; the latter is referred to as “scarce” hereafter. This dataset was used as training data in the present study, whereas the new data collected under commercial farming conditions served as test data. The test dataset excluded cows in heat, because estrus is known to affect feed intake as well as behavior of dairy cows [29]. Besides, in commercial farming, animal caretakers are often aware of a cow’s estrus and know how to interpret the associated behavioral changes. Moreover, some observations had to be omitted, because data on one or several of the eight behavioral variables, the milk yield, or rumen fill scores were missing. In total, 583 cow-days served as test data.

For the test dataset (provided in File S1), the herbage availability classes 0 and 1 were predicted using both approaches, GLM and RFM. The analysis was performed using the statistical computing language R (Version 4.0.3, R Foundation, Vienna, Austria). Therefore, the functions “glm” of the R-package “stats” and the function “randomForest” of the R-package “randomForest” were applied as described by Shafiullah et al. [19,40,41]. The binary classification, i.e., the response variable, was based on the probability level 0.5, such that if $\hat{p}$ < 0.5, y = 0 (i.e., negative class indicating sufficient herbage availability), and if $\hat{p}$ > 0.5, y = 1 (i.e., positive class indicating scarce herbage availability).

### 2.9. Evaluation of Prediction Models

#### 2.9.1. Predictive Performance

To evaluate the performance of the binary classification, model predictions were compared to both reference indicators, the herbage availability classification derived from the milk yield recordings and from the rumen fill scoring, as well as the combination of both reference indicators. For the latter, cow-days with either a significant milk yield (positive class = 1) or rumen fill reduction (positive class = 1) detected on days 3, 4, 5, or 6 were classified as positive. The remaining cow-days were classified as negative (=0).

With this, confusion matrices were created using the R package “caret” [42], where prediction classes and reference classes (i.e., true) were contrasted to identify true positives (TP), true negatives (TN), false positives (FP), and false negatives (FN). Thereafter, the sensitivity (i.e., ratio between TP and the sum of TP and FN), the specificity (i.e., ratio between TN and the sum of TN and FP), and the positive predictive value (PPV; i.e., ratio between TP and the sum of TP and FP) were calculated. Specificity and sensitivity were measures of the relative number of the true negative and true positive cases a model was able to detect. In contrast, the PPV indicated how many cases predicted as positive were actually positive cases. Finally, the area under the receiver operating characteristic curve (AUC) was determined using the R package *caret* to obtain a measure for model accuracy when corrected for imbalanced class distribution. Binary classifications with an AUC < 0.5 were assumed to indicate a failing performance, because 0.5 describes a random classification [20]. AUC values of 0.5 to 0.6 were considered to indicate a weak performance, whereas those greater than 0.6 indicated an even more viable model performance.

In order to assess the performance of the prediction models in different animals more accurately, several cow subgroups were formed based on their characteristics: parity, grazing experience from previous years, milk performance, gestation stage, and body condition (Table 3). Classification limits for milk performance and BCS were chosen to balance the sample size per class as much as possible. The assignment to a subgroup was based on the cows’ characteristics at the beginning of each grazing cycle (i.e., for milk performance, gestation stage, and BCS) and did not change within a six-day grazing cycle.

Lastly, the performance in predicting scarce herbage availability of the reference classification created from milk yield recordings was investigated. To do so, the classification derived from milk yield recordings was compared to the reference classification derived from rumen fill scoring. The same performance metrics were calculated as described above.

#### 2.9.2. Variable Importance

To visualize the effects of the gradually decreasing herbage availability on pasture over the six grazing days on cow behavior, a mixed effects model was constructed using the R packages *nmle* and *splines*. Within the function *lme* [43], cow ID and paddock ID were random effects for intercept and slope, respectively. The day within each grazing cycle (six factor levels: days 1, 2, 3, 4, 5, and 6) was a fixed effect specified as natural cubic spline. For the natural cubic splines, five degrees of freedom were used [44]. As a result, the model included four internal knots as well as two boundary knots at days 1 and 6. The predicted splines were plotted in combination with violin plots along with the lower and upper 95% confidence intervals. Behavioral variables that did not indicate a relevant change in one direction, i.e., either decrease or increase, during the last four grazing days were identified as predictors of low relevance. They were then removed as predictors, and the predictive performance of the models was retested as described above.

The R package *ggplot2* and the function *geom_violin* were used to present data in violin plots [45]. With their width, the violin plots indicate the probability density of the data at certain values.

**Figure 2:**
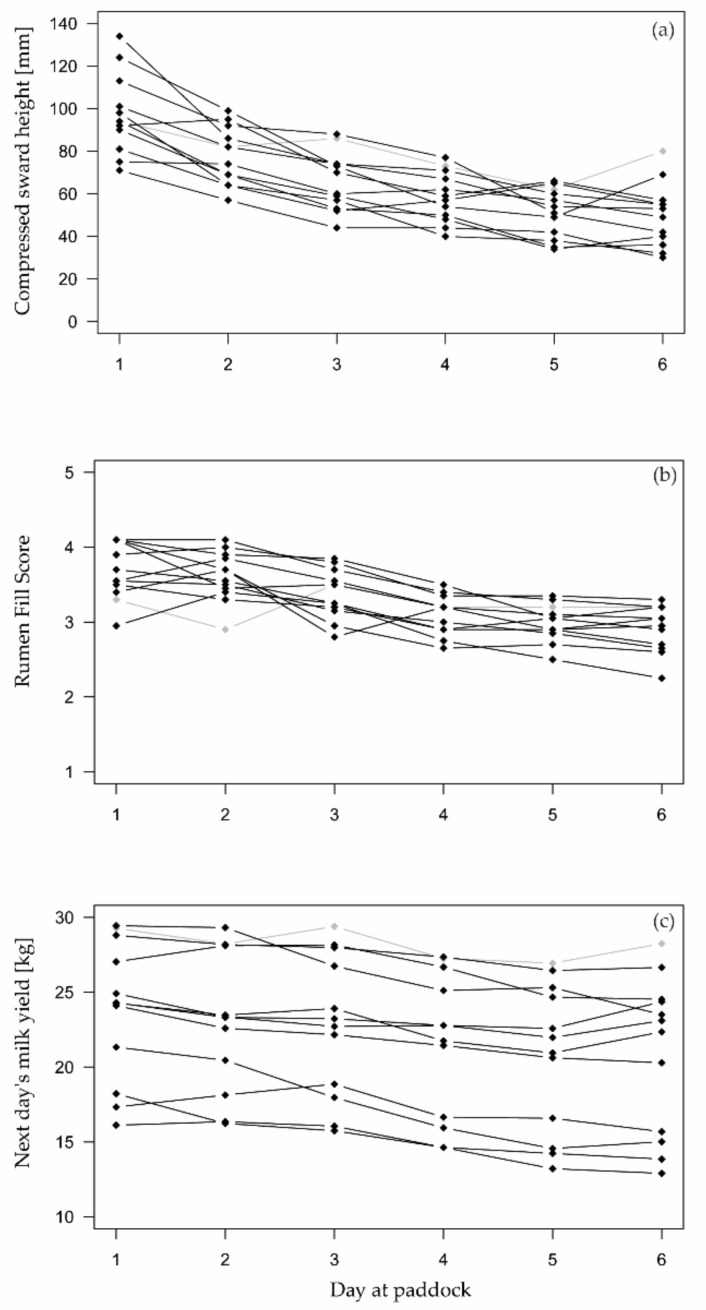
Mean post-grazing compressed sward heights per paddock (**a**), mean rumen fill score across 20 cows (**b**), and mean next day’s milk yield (recorded on days 2 to 7 as indicator for herbage availability on days 1 to 6, respectively) across 20 cows (**c**) at each grazing day, respectively. Lines between points connect measurements that belong to the same grazing cycle and do not visualize a regression analysis. Highlighted in gray is Paddock 1, which was excluded for the analysis in Section 3.3.

For the analysis of variable importance (i.e., changes in behavioral variables as a response to gradually declining herbage availability), Paddock 1 was excluded (highlighted in Figure 2), because cows might have avoided grazing due to heavy rains on day 3 that had covered herbage with soil.

## 3. Results

### 3.1. Effects of the Paddock Allocation

During the six-day grazing cycle, the available herbage on pasture decreased from an average pre-grazing CSH of 125 mm (SD: 24) to an average post-grazing CSH of 50 mm (SD: 14) at day 6 (Figure 2). At that day, the CSH ranged from 30 mm to 67 mm, except for Paddock 1 (Table 1), where post-grazing CSH was 80 mm.

Both reference indicators, which were used to identify scarce herbage availability, changed noticeably across all cows during the six grazing days (Figure 2). Milk yields decreased on average from 23.2 kg (SD 6.8) to 20.2 kg (SD 6.4) over six days, while rumen fill scores declined from 3.7 (SD 0.59) to 2.9 (SD 0.59) from day 1 to day 6, respectively.

### 3.2. Herbage Availability According to Reference Indicators

**Figure 3:**
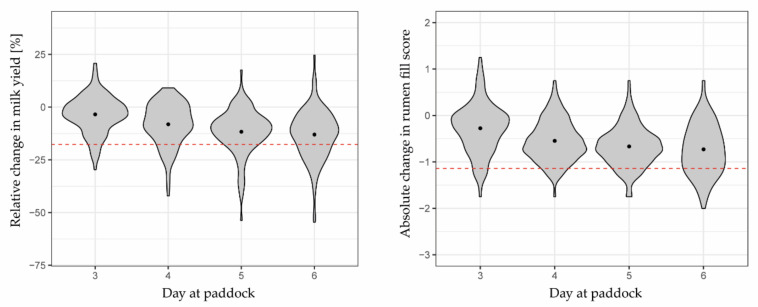
Violin plots of reference indicators showing relative changes in milk yield (**left**) and absolute changes in rumen fill scores (**right**) from grazing days 3 to 6, compared to baseline values at 0%. Points indicate means across 20 cows. The width of the violins indicates how high the probability density is at the given value of the y-axis. Any change below the red line was classified as 1 (i.e., scarce herbage availability), whereas any change above the line was classified as 0 (i.e., sufficient herbage availability). The presented milk yields were recorded one day later than indicated in the graph as indicators for the herbage availability on the previous grazing day.

On average, milk yields and rumen fill scores steadily declined until day 6 (Figure 3). On that last day of grazing at paddock, average milk yield and average rumen fill score decreased by 10.4% (SD 13.5%) and 0.73 (SD 0.54), respectively, compared to their baseline values on days 1 and 2. However, even on day 6, decline in milk yield and rumen fill score was not always above the limit to classify a cow-day as “scarce”. Thus, there was a relatively small number of positive reference classes in the test dataset.

With the limit in the reduction in daily milk yields of 17.7% of the baseline value, a total of 72 cow-days were classified positive, indicating scarce herbage availability (Table 4). Using the reference indicator of rumen fill scoring with the reduction of 1.14 as limit, there were 19 cases fewer (53 cow-days). Both reference indicators agreed in 24 cases; hence, combining both resulted in 101 out of 583 cases being positive (17.3%). Nevertheless, with all three measurements, the number of positive cases increased towards day 6 (Table 4), from 12.9% of the cow-days on day 3 to 42.4% of the cow-days classified as “scarce” on day 6 across all grazing cycles when both reference indicators were combined.

### 3.3. Behavioral Changes

**Figure 4:**
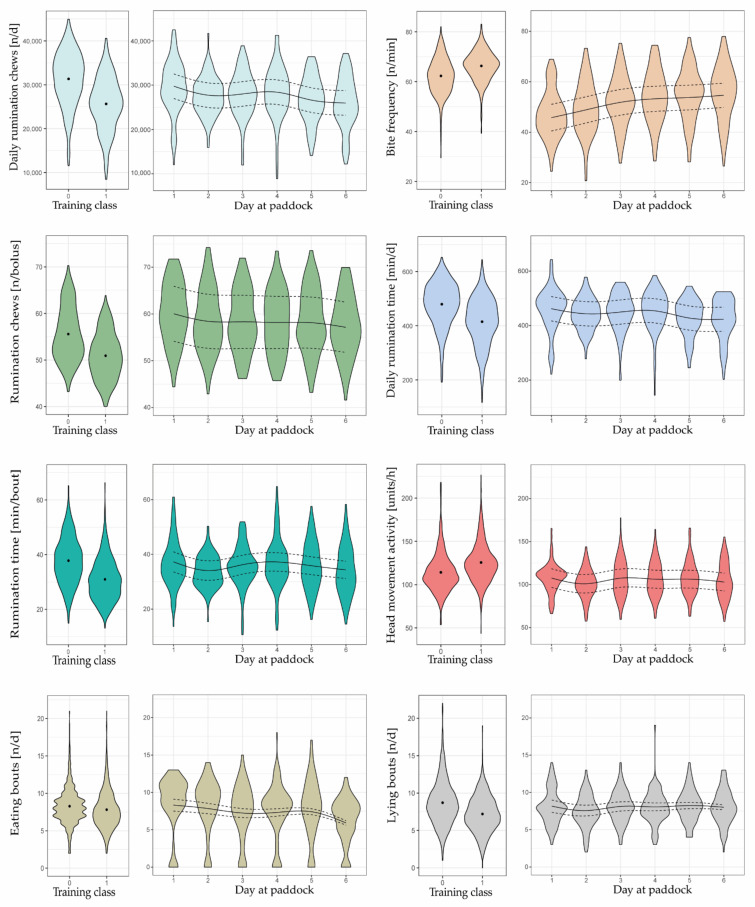
Violin plots showing differences in animal behavior between the feed status classes 0 (i.e., sufficient) and 1 (i.e., scarce) of the training dataset as well as differences between grazing days 1 to 6 across six grazing cycles and 20 cows of the test dataset. The points indicate mean values. The lines indicate estimates of the mixed effects model with splines (solid) along with the upper and lower 95% confidence intervals (dashed). The width of the violins indicates how high the probability density is at the given value of the y-axis.

Compared to the training data, mean bite frequencies were lower in the test data, and mean number of rumination chews per bolus tended to be greater (Figure 4).

During the six days at paddock, some of the eight behavioral variables changed in a relevant manner as herbage availability gradually decreased, indicating their importance in predicting herbage scarcity. All variables indicated a visible decline from day 1 to day 2, except the frequency of eating bites, which increased. This change in behavior was due to ad libitum feeding of meadow hay on day 1.

Mean bite frequency, estimated by the mixed effects model with splines, continued to increase from day 2 (48.8 bites min^−1^) to day 6 (54.6 bites min^−1^), whereas the increase was pronounced from day 2 to day 3 (+3.0 bites min^−1^) but very small during subsequent days (+1.4, +0.5, and +0.9 bites min^−1^). At the same time, the number of eating bouts, the daily number of rumination chews, the rumination time per bout, and the daily rumination time tended to decrease as days at paddock progressed, particularly towards days 5 and 6 of each grazing cycle (Figure 4). Conversely, the head movement activity and the number of lying bouts did not change in a clear direction.

The greatest decline in the number of eating bouts was observed from day 5 to 6 (−1.5 n d^−1^). Daily rumination chews and rumination time decreased greatly from day 4 to 5 (−1916 n and −26.5 min d^−1^, respectively). Similarly, rumination time per bout declined pronouncedly from day 4 to 5 (−1.5 min bout^−1^) as well as from day 5 to 6 (−1.5 min bout^−1^).

The number of rumination chews per bolus decreased slightly over days 3 to 6, but it was not until day 6 that a slight change was seen (−1.0 n bolus^−1^ compared to day 5). Nonetheless, this variable, along with the head movement activity and the number of lying bouts, was irrelevant in predicting a gradually decreasing herbage availability. This is supported by the fact that the variables did not change relevantly during the last four grazing days; they neither increased nor decreased.

**Figure 5:**
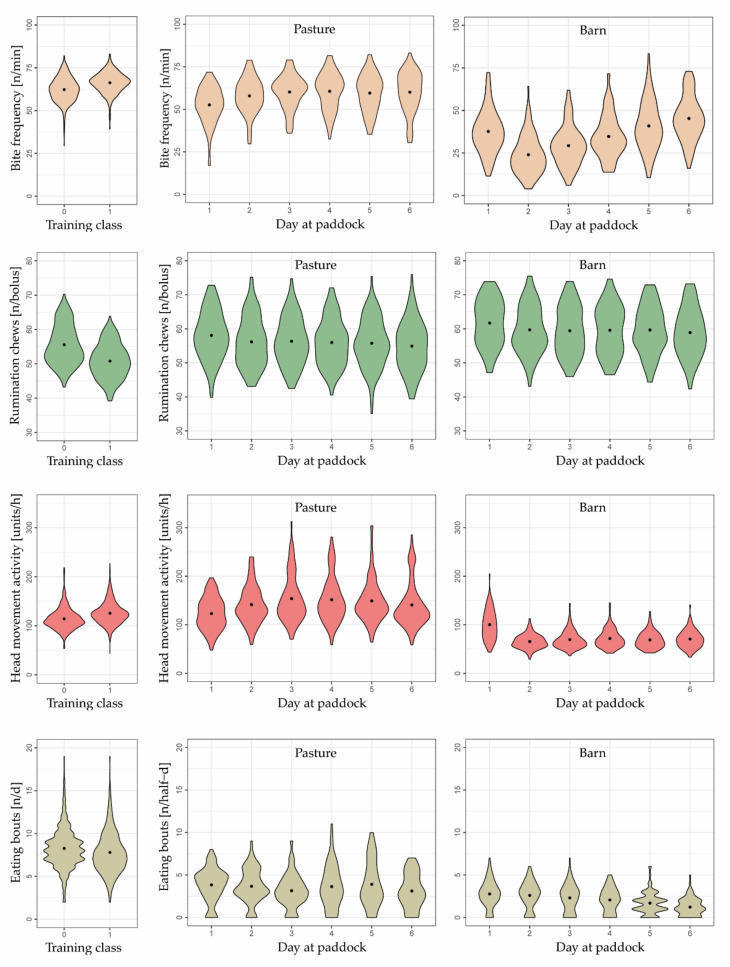
Violin plots showing differences in animal behavior between the feed status classes 0 (i.e., sufficient) and 1 (i.e., scarce) of the training dataset as well as differences during the hours on pasture or in the barn from days 1 to 6 across six grazing cycles and 20 cows of the test dataset. The points indicate mean values. The width of the violins indicates how high the probability density is at the given value of the y-axis. Data of other variables are shown in Figure A2.

When data were analyzed separately for the time cows spent on pasture or in the barn, bite frequency and number of eating bouts during the time in the barn changed more pronouncedly with advancing grazing cycle than during the time on pasture (Figure 5). Conversely, head movement activity during the time in the barn was similar across days 2 to 6 but increased continuously until day 4 and then decreased until day 6 during the time on pasture.

**Figure A2:**
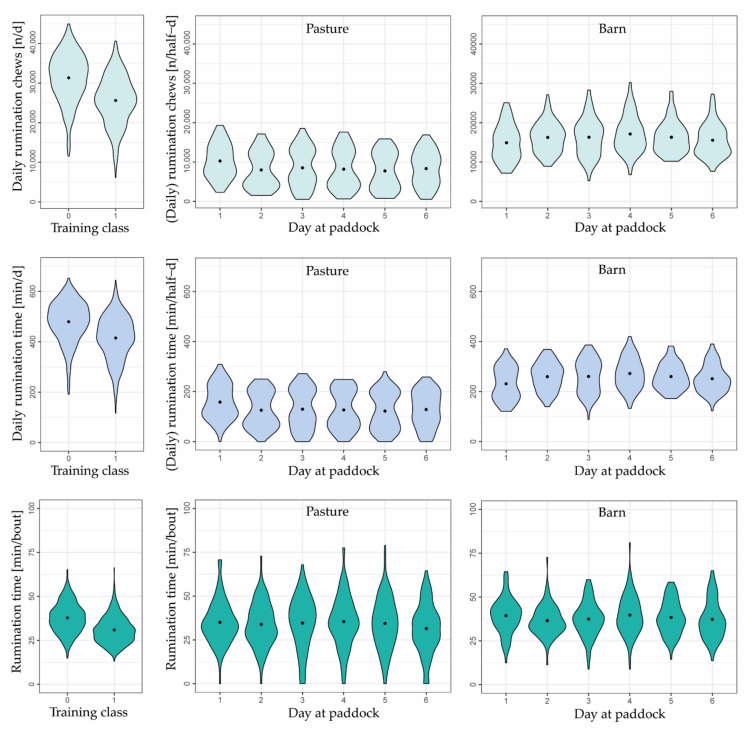
Violin plots showing differences in animal behavior between the feed status Classes 0 (i.e., sufficient) and 1 (i.e., scarce) of the training dataset, as well as differences during the hours on pasture or in the barn from days 1 to 6 across six grazing cycles and 20 cows of the test dataset. The points indicate mean values. The width of the violins indicates how high the probability density is at the given value of the y-axis. Data from other variables are shown in Figure 5, except for the variable “lying bouts”, where data could not be split into hours on pasture and in the barn, because it was recorded as a daily sum.

Rumination-related variables, which were calculated as sums within a certain time interval (rumination chews and rumination time per half-day), often changed in an opposite pattern with the period on pasture in contrast to the period in the barn (Figure A2). The ranges of these two variables and of eating bouts were only within the range of the training dataset when barn and pasture periods were summed up.

### 3.4. Predicted Herbage Availability

With the underlying training dataset, the two prediction approaches, GLM and RFM, classified a total of 57 and 45 cow-days, respectively, as positive, i.e., scarce herbage availability (Table 5), equivalent to 9.8% and 7.7% of the total cow-day observations. These proportions are approximately in line with the number of cases identified as “scarce” by both reference indicators (9.1% and 12.3%; Table 4). In both prediction approaches, no cow was classified as “scarce” on day 1. In the case of the GLM, the number of positive cases increased continuously towards day 6. For the RFM approach, the number of positive cases remained almost identical across days 3 to 5 and increased on day 6.

### 3.5. Predictive Performance

Model predictions best matched classifications using rumen fill scoring as a reference classification (Table 6). Specificity of the prediction models, i.e., the ability to correctly identify herbage sufficiency, was high (0.90 to 0.92), irrespective of the prediction approach (i.e., GLM or RFM). In contrast, sensitivity and PPV were poor (0.06 to 0.15 and 0.07 to 0.21, respectively).

In general, GLM performed slightly better than RFM before the number of predictors was reduced. Reducing the number of predictors from eight to five relevant behaviors slightly improved the models’ performances, especially their sensitivity (Table 6). Both models, GLM and RFM, performed similarly after variable reduction.

Using rumen fill scoring as the reference classification, milk yield predicted herbage availability with a sensitivity of 0.45, a specificity of 0.91, a PPV of 0.33, and AUC of 0.68. The fact that these performance metrics are higher than the metrics resulting from GLM and RFM predictions compared to the rumen fill reference classification indicates that milk yield recordings outperformed the prediction of herbage availability based on behavioral variables.

Comparing different subgroups of animals, predictive performance of both models was greater in high-performing (>27 kg d^−1^) than in medium- or low-performing cows; in multi- than in primiparous cows; in cows with low BCS (≤2.5) than in cows with medium and high BCS; in cows in gestation than in empty cows; and in cows with grazing experience from previous years than in cows without grazing experience (Table A2). Thus, changes in behavior of the above-mentioned animal groups were more congruent with a decrease in their milk yield and rumen fill score. Using the GLM approach with five predictors and comparing it to the classification where reference indicators were combined, performance for the animal groups rose to AUC ≥ 0.56, sensitivities to between 0.26 and 0.47, and PPV to between 0.19 and 0.36. The highest AUC values were obtained for cows with low body condition scores (0.60 and 0.67 with eight and five predictors, respectively) and high-performing cows (0.57 and 0.56 with eight and five predictors, respectively).

## 4. Discussion

Both modeling approaches for predicting scarce herbage availability performed more poorly in the present evaluation study under commercial farming conditions than they did during model development, where they reached an AUC of 0.76 for both GLM and RFM. The sensitivities were 0.74 and 0.75 and PPVs 0.63 and 0.60 using leave-one-out-cross-validation [19]. Therefore, GLM and RFM including the eight selected variables proposed by Shafiullah et al. [19] were not generalizable to the grazing set-up in the present study using the underlying training dataset. This shows that the prediction models for herbage scarcity based on behavioral changes cannot yet be applied to various grazing and pasture conditions, feed supplementation levels, or cow breeds without impairing model performance.

Thus, we discuss different possible sources that may have contributed to low model performance before providing an outlook for further model-based decision support in allocating new pasture at the end of this section.

### 4.1. Differences between Training and Test Data as a Cause for Low Model Performance

The main differences between the training and testing experiments were, firstly, the level of feed restriction on pasture; secondly, the grazing set-up (i.e., timing of allocating new pasture and the hours spent daily on pasture and in the barn); and, lastly, the choice of reference measurements.

The first two points mentioned could have affected the response of some behavioral variables to declining herbage availability over the six grazing days and, thus, their contribution to a correct prediction of herbage scarcity. In the present experiment (testing), allowances of herbage and supplement feed were reduced by 20%, whereas a reduction in the daily feed intake by 40% was targeted in the Irish experiment (training) used as a basis for model development [17,19]. The restriction level of 20% was intentionally chosen to ensure animal welfare and to mimic practical situations in which farmers should notice and react early in the event of herbage scarcity. Nevertheless, the lower level of feed restriction in the present experiment might be the reason why, in some behavioral variables, no clear changes were visible with decreasing herbage availability when looking at the 24 h data (Figure 4). However, differences in the grazing set-up likely also played a role, because the reference indicators did respond to declining herbage availability (Figure 3) and clearly indicated herbage scarcity (Table 4).

The grazing set-up in training and testing experiments differed in the timing of when cows were allocated to new pasture area and in the time they spent on pasture each day.

During the training experiment, cows were on pasture full-time, and a new strip was allocated twice daily after milking, albeit with the defined herbage restriction [17]. Thus, the animals were able to graze fresh but very limited amounts of herbage which, additionally, declined during the following hours. In contrast, during the present testing experiment, one grazing cycle lasted six days before a new paddock was allocated so that the CSH decreased with each additional day (Figure 2a). Therefore, cows did not have access to fresh herbage each day but went to a pasture with less and less herbage mass left.

In addition, the animals were out on pasture only half the day and in the barn the other half of the day, where they were fed a restricted amount of meadow hay once daily. This feed allocation in the barn was comparable to the allocation of a new strip of pasture. The effects of allocating new feed on the animals’ behavior are presented in Figure 5, which shows the 24 h data separately for the time on pasture and in the barn.

Changes in the behavior of cows on pasture might have often been masked by their behavioral responses during the time in the barn and vice versa when analyzing the animals’ responses to gradually declining herbage availability on a 24 h basis including both the time on pasture and in the barn. In this line, some behavioral variables relevant to the daily prediction of herbage scarcity in the Irish training dataset responded more pronouncedly in the present test dataset when looking only at the behavior during the time on pasture (i.e., head movement activity) or in the barn (i.e., increasing bite frequency and decreasing eating bouts).

Moreover, the ranges of values in the behavioral variables differed between the training and test datasets, which could have affected the predictive performance of the models. For example, on day 6, bite frequency (n min^−1^), head movement activity (units h^−1^), and rumination chews per bolus (n bolus^−1^) were still closer to the mean values of Training Class 0 than to those of Training Class 1 (Figure 4). The importance of these three variables was below that reported in Shafiullah et al. [19] (identified with variable importance plot of the RFM, results not shown). Interestingly, in the present study, the behavioral variables contributed less to the correct prediction than what was expected by Shafiullah et al. [19].

All three variables were calculated as a value per hour, using the RumiWatch Converter, and averaged across the day. The bite frequency and the head movement activity were often below and the rumination chews per bolus above those of the training dataset (Figure 4). In contrast, values related to the three behavioral variables were closer to those of the training dataset when considering only the grazing phase, eliminating data from the barn phase (Figure 5). This is plausible, because behavior of cows in the barn differs considerably from that on pasture, which is due to the finite amount and structure of the feed in the barn (see also Section 4.3), and, thus, mean values for the whole 24 hours were distorted. In the end, however, it also means that some behavioral variables on a 24 h basis generated from a part-time grazing system cannot be compared to 24 h summaries from a full-time grazing system. This leads to the assumption that some variables are better suited for developing a robust decision support algorithm than others.

Finally, another difference between training and testing experiments was in the reference measurements to distinguish between sufficient or scarce herbage availability. In the Irish experiment, a parallel group design was established to generate balanced training data for model development [17,19]. There, daily herbage allowance for the control group (*n* = 10 cows) and the group subjected to 40% herbage restriction (*n* = 30 cows) could be more precisely adjusted by moving the fences, compared with the herbage allowance in a six-day allocation set-up. Therefore, the reference classification was much clearer in the Irish experiment. In contrast, in the present experiment, it was more challenging to reveal less pronounced day-to-day changes in DM intake of individual animals. The chosen reference indicators were able to fulfill this objective and proved their practicability as indicators in farming practice. However, the formulation of their thresholds for a change in the binary classification certainly played a role in the model evaluation.

### 4.2. The Challenge of Evaluating the Binary Predictions with Gradually Changing Reference Indicators

In our grazing set-up, a decline in milk yield and a reduced rumen fill score beyond the normal variation were used as proxies for scarce herbage availability. Both reference indicators were chosen because they have the great advantage of responding rapidly to variations in DM intake [30,31,46], can be easily measured on commercial farms to support herd management [5,47], and, thus, can potentially be used to generate training data on-farm.

However, both reference indicators are subject to uncertainties with respect to the study objective. Milk yield and rumen fill could also be reduced by factors other than a scarce herbage allowance. For example, milk yield may also be reduced in the short term once estrus sets in [29] or as a result of reduced feed intake and/or an increased activity and energy expenditures caused by diseases [48,49], which would also affect rumen fill [31,47]. However, data of cows on days where signs of estrus or illness were observed were excluded from the dataset in the present study. Therefore, a decline in milk yield and a reduction in rumen fill was most probably due to decreasing herbage availability on pasture.

Another uncertainty is the agreement of the binary classification resulting from the model predictions with the binary classification by the reference indicators, because the exact response time of both reference indicators, i.e., the duration until the indicator changes after a feed challenge, and the degree of response are uncertain.

For rumen fill, studies have shown that the score directly reflects the level of feed intake during the previous two to six [30], or even eight, hours [47]. Rumen fill scoring was, therefore, the more timely and direct indicator for the amount of feed consumed by a cow in the present study, compared to milk yield, where metabolic processes might have influenced the response time and response degree to a previous feed challenge [50].

Daily milk yield in the present study often decreased gradually and with a delay of one day, because it had recovered almost completely at the day after feeding ad libitum, which was done immediately at the end of the first grazing cycles. Similarly, Herve et al. [46] showed that daily milk yield decreases directly upon a feed restriction to 80% of the cows’ ad libitum DM intake but not, however, to the full extent, as it continued to decrease with continued feed restriction [46]. The trend, over days, of the milk reduction played an important role in our experiment, because, depending on which day the milk yield of one cow reached the pre-defined threshold, the classification changed from 0 to 1.

If this change from Class 0 to 1 did not occur on the exact same day as predicted by the GLM and RFM, there were FPs and FNs that depressed model performance. Likewise, Post et al. [20] and Post et al. [51] highlighted the challenge of detecting the correct time points of occurrence of rare events with a binary system; in their studies, the first signs of lameness and mastitis were already detectable in behavioral data a few days before being diagnosed by a person, which resulted in a high number of FPs.

Consequently, we may be able to detect diseases, or in the present case herbage scarcity, earlier with behavioral data than by the reference methods, but we may evaluate the developed models to be worse than they are.

In addition, it would be useful if models would consider the trends of behavioral changes between days on an individual-animal basis. So far, using Shafiullah’s models, the same animal could have been classified as 1 on day 4 and again Class 0 on day 5. Therefore, future models should consider day-to-day correlations within one cow.

A third challenge for the modeling approach is individual animals in which behavior or reference indicators do not change even though herbage availability declines.

If a cow maintains its normal level of milk yield even when herbage availability on pasture declines, it would not be classified as “scarce” (i.e., Class 0) by the milk yield reference indicator. Nevertheless, the cow may have been restless and intensified its foraging activity (i.e., less lying down and rumination activity as well as increased bite frequency and head movement activity), causing it to be identified as “scarce” (i.e., Class 1) by GLM and RFM.

One explanation could be that cows which did not show any decline in milk yield could be animals who are more adaptable than others in terms of nutrient partitioning to support lactation [50] or showed more grazing perseverance to the point of possibly even consuming the required amount of feed from pasture with little herbage mass left. Another reason could be that those cows have generally lower feed requirement (and presumably produce less milk) but continue grazing, even though feed is not required.

The said potential sources of error resulted in a great number of FPs (50 out of 583 cow-days in the case of using GLM and the milk yield reference indicator; Table A3) and, thus, in a low model performance.

Not only was the number of FPs relatively high in our study, but the number of FNs was too (65 out of 583 cow-days in the case of using GLM and the milk yield reference indicator; Table A3). False negatives are days when a cow produced noticeably less milk or had a lower rumen fill than usual (i.e., Class 1) but did not exhibit the characteristic behavior of herbage scarcity as found by Werner et al. [17] and Shafiullah et al. [19], namely, increased restlessness and intensified foraging behavior (i.e., Class 0). These are likely animals with low plasticity to adapt to changing circumstances. Such days will remain FNs in any future usage of the prediction model and, thus, will reduce the sensitivity of the models.

Low sensitivity values occurred mainly in the animals with lower levels of milk production and better body condition (Table A2) and, presumably, in cows with lower levels of feed intake [50]. These cows had enough body reserves, and there was no need to change behavior in order to have sufficient feed intake for maintenance. Therefore, it might be useful to include milk yield recordings, although somewhat delayed in its response to reduced herbage availability, as an additional predictor in the model [52,53].

Furthermore, it is essential to base pasture allocation decisions on a percentage of the herd rather than one animal. The individuals in focus should be cows that produce high milk yields and have lower BCS, because they have greater feed intake levels and greater energy requirements [50], and it is likely that they respond more sensitively to declining herbage availability (Table A2).

### 4.3. Suitability of Behavioral Variables as Predictors Depends on Grazing Condition

Previous studies found decreases in lying bouts [16], eating bouts [54], and rumination time [55] and an increase in prehension bite frequency [56] and head movement activity [19] in response to gradually declining herbage availability. Correspondingly, we expected similar changes in the behavior of cows from days 1 to 6. These expected changes in animal behavior appeared in five variables, namely, the daily rumination chews, bite frequency, daily rumination time, rumination time per bout, and eating bouts. However, the expected behavioral changes were ambiguous in three variables (i.e., rumination chews per bolus, head movement activity, and lying bouts) where no pronounced and stepwise changes across two or three days were visible, such that they were not suitable predictors for a binary classification approach. Therefore, the question arose whether these behavioral variables are suitable predictors of scarce herbage availability in general and under which grazing conditions and supplementation schemes they allow for accurate prediction.

Both the head movement activity and the number of lying bouts did not change notably across the last four days of a grazing cycle. Similarly, rumination chews per bolus remained relatively constant until day 5 and then decreased only minimally (Figure 4). We suspected that these three behavioral variables might not be important as predictors of herbage scarcity in a part-time grazing system, at least not when evaluated on a 24 h basis (see Section 3.3), because model performance rose when we excluded them from the list of predictors (Table 6). However, after separating the 24 h data into the hours on pasture and in the barn, it became clear that a behavioral change in response to declining herbage availability on pasture was present in some variables, but especially in the head movement activity, masked by the exhibited behavior during the time spent in the barn (Figure 5). Thus, the variables measured on a 24 h basis were less sensitive to changing conditions on pasture than expected, because they included the half-day phase in the barn. In the barn, the cows could not exhibit an intensive foraging behavior and restlessness with high head movement activity, because, at a certain point in time, the limited amount of feed at the feeding bank was eaten. Presumably, a main part of the remaining time was used to stand and to rest, to lay down, and to ruminate.

Contrarily to head movement activity, the number of eating bouts decreased even more strongly in the barn than on pasture. Werner et al. [17] explained the decreasing number of eating bouts under herbage scarcity by the fact that the duration of the bouts was longer in order for cows to find the required amount of herbage. In the present experiment, the decreasing number of eating bouts in the barn can be explained by the fact that, with declining herbage availability on pasture, the limited amount of meadow hay allocated in the barn was increasingly consumed in one piece, with a decreasing number of interruptions.

For future development of the approach, the variable “head movement activity” could be a suitable predictor when considering only behavior exhibited on pasture. Although it responds in a non-linear way, its vertex carries important information, because the variable increases as long as there is still new feed to be found in the pasture, and the vertex emerges presumably at the time when most new leaf mass within close range is consumed and cows must intensify foraging behavior (day 3 or day 4, Figure 5).

Consistent with the findings of Kennedy et al. [56], Figure A2 and Figure 5 show that rumination mainly occurred during the time spent in the barn, and the hours spent on pasture were used for foraging. To consider this compensation, in future applications of the model in part-time grazing systems, the rumination-related variables, representing a daily sum, should address both the grazing and barn phases. However, the variable “rumination chews per bolus” might be excluded in future applications of the modeling approach. This is not only because the variable did not change relevantly across the six-day grazing cycle, but because, already during model development, Shafiullah et al. [19] found that the variable, which ranged from around 40 to around 70 n bolus^−1^, could not be assigned to one of the two classes in the range between 50 and 60 n bolus^−1^, indicating that the variable is less suitable as a predictor in a binary classification problem.

The average frequency of prehension bites increased clearly over the six-day grazing cycle when looking at the 24 h data (Figure 4) and was already identified as a very relevant predictor in previous studies [17,19]. However, in the present grazing set-up, the variable was even more sensitive to declining herbage availability during the time in the barn than on pasture (Figure 5). This could be explained by the fact that there was a limited amount of new feed allocated each day, comparable to the strip-grazing conditions in the training experiment, and that feed was eaten more and more greedily towards the end of the six-day grazing cycles when the cows returned from the pasture. This suggests that the variable “bite frequency” is definitely suitable as a predictor in a grazing system with an allocation of new feed or pasture strips, and changes in bite frequency should be taken into account by focusing on the time span when new feed is allocated and not when cows are actually on pasture with declining herbage availability.

### 4.4. Outlook

In order to develop future commercial decision support tools, they must be suitably robust to system differences [6,20]. In the present experiment, the prediction models, which used 24 h measurements of cow behavior to detect scarce herbage availability, did not perform satisfactorily with the underlying training data. Thus, the evaluated model approach did not fit sufficiently to a more typically Swiss grazing system, a six-day rotational grazing system under half-day barn conditions. However, the data analysis has provided a useful input. We identified three key points which can be put into context for further model development as follows:

Firstly, the bite frequency and the daily number of rumination chews are confirmed to be important variables, as already identified by Shafiullah et al. [19]. However, bite frequency needs to be seen in the context of new feed allocation, because it increased with scarce herbage availability, particularly strongly during allocation of new pasture strips in the study of Shafiullah et al., and during supplementary feeding in the barn in the present study. Therefore, the aspect of new feed allocation within the grazing system holds important information regarding possible herbage scarcity [57].

Secondly, data analysis should focus on a higher resolution than 24 h summaries. Authors working on similar topics, such as Paulenz et al. [58], also suggested analyzing shorter time spans, in the context of automated behavioral monitoring, for an estimate of herbage availability on pasture instead of analyzing 24 h summaries.

Thirdly, a future decision support tool for pasture allocation scheduling based on animal behavior could include milk yield recordings [52,53]. In general, for binary classification models, it is the case that the more a predictor is related to the classification variable, the better is the AUC [51]. For example, milk yield is known to be very important in predicting the feed intake of cows [53,59]. Our results showed that milk yield recordings, which were used as reference indicator in the present study, have potential as a reliable predictor of herbage availability on pasture. However, milk yield has a postponed response [60] of roughly one day [46], which might delay the warning for herbage scarcity.

On the contrary, the reference indicator based on rumen fill responds relatively promptly to changing feed intake [30,31]. Thus, as a reference indicator, rumen fill is more favorable than milk yield. However, milk yield is automatically recorded so that it can be a useful additional source of information for the model.

Summarizing this chapter, there is scope to improve the modeling approach to identify herbage scarcity on pasture and to provide farmers with a more robust tool to support their decision making in allocating new pasture.

## 5. Conclusions

The prediction models showed poor performance under the applied grazing conditions, but this increased when insensitive predictors (rumination chews per bolus, number of lying bouts, and head movement activity) were excluded. Moreover, daily rumination chews and the (prehension) bite frequency are confirmed to be suitable predictors, as evaluated in previous studies.

There is potential for some of the behavioral predictors to indicate herbage scarcity and to further develop this approach according to different grazing set-ups. For example, in order to predict herbage scarcity on a daily basis, the predictor “bite frequency” is particularly sensitive to gradually declining herbage availability on pasture when new feed is allocated. Following this train of thought, to predict herbage scarcity on the basis of behavioral changes, it is important to provide either new herbage on pasture or feed supplementation in the barn at least once a day in order to identify the characteristic changes in bite frequency. If the system aims for a longer-duration grazing set-up, we recommend training a separate model which might include differing predictors, as the characteristic bite frequency changes do not occur under such circumstances. Furthermore, we identified the head movement activity during hours of grazing as an additional meaningful predictor for the time span on pasture, because the vertex emerges before the rumination-related variables respond to declining herbage availability. Therefore, the potential decision support tool could issue a warning before a drop in milk yield occurs.

## Figures and Tables

**Table 1:** Features of the allocated paddocks and the available herbage mass.

Number of Grazing Cycle and Month	Cow Group	Paddock ID	Paddock Area (ha)	Pre-Grazing CSH (mm)	Allocated HM at Beginning of Grazing Cycles (kg DM Group^−1^ Paddock^−1^) *
1–May	A	1	0.52	93	679 ^#^
1–May	B	2	0.31	117	534
2–May	A	3	0.19	164	496
2–May	B	4	0.19	162	494
3–July	A	5	0.34	89	476
3–July	B	6	0.29	104	493
4–July	A	7	0.25	125	509
4–July	B	8	0.27	113	500
5–September	A	9	0.17	152	569
5–September	B	10	0.19	125	564
6–September	A	11	0.20	120	564
6–September	B	12	0.19	131	565

ID: identification code, CSH: compressed sward height, HM: herbage mass, DM: dry matter. * Estimated with calibrations (Table A1) for herbage above 20 mm CSH. ^#^ Excluded from the analysis in Section 3.3.

**Table 2:** Behavioral variables used in the present study (adapted from Werner et al. [17] and Shafiullah et al. [19]).

Variable (Unit)	Corresponding Variable Name *
Daily rumination chews (n d^−1^)	RUMINATECHEW
Bite frequency (n min^−1^), represents the prehension bites with head down during eating	BITEFREQ
Rumination chews (n bolus^−1^)	RUMICHEWBOLUS
Head movement activity index (units h^−1^), calculated from acceleration in x, y, and z directions	HACTIVITY
Rumination time (min bout^−1^)	RUMIBOUTLENGTH
Daily rumination time (min d^−1^), calculated as the sum of rumination bout minutes	RUMIBOUTTIME
Eating bouts (n d^−1^)	GRAZINGSTART
Lying bouts (n d^−1^)	LAYDOWN

* As given by the RumiWatch Converter and as published in Shafiullah et al. [19].

**Table 3:** Features of the formulated subgroups of cows.

Subgroup	Splitting Criteria	Number of Cow-Days (*n*)
Primiparous cows	Parity = 1	209
Multiparous cows	Parity > 1	374
Cows with grazing experience	Cows grazed in previous years	274
Cows without grazing experience	Cows never grazed previously	309
High-performing cows	Milk yield > 27 kg d^−1^ (mean: 31.6)	186
Medium-performing cows	Milk yield 20–27 kg d^−1^ (mean: 23.8)	212
Low-performing cows	Milk yield < 20 kg d^−1^ (mean: 16.5)	185
Empty cows	Gestation verified	220
Cows in gestation	Gestation not verified	363
High BCS	BCS ≥ 3.25	219
Medium BCS	BCS 2.75–3.00	223
Low BCS	BCS ≤ 2.5	141

BCS: body condition score.

**Table 4:** Number of cow-days with scarce herbage availability identified by the reference methods on days 1 to 6 across six grazing cycles for two groups of 10 cows each. The number of total observations per grazing day is provided in parentheses.

Reference Methods	Day 1 ^+^ (*n* = 91)	Day 2 ^+^ (*n* = 92)	Day 3 (*n* = 101)	Day 4 (*n* = 101)	Day 5 (*n* = 99)	Day 6 (*n* = 99)	Total (*n* = 583)
Milk yield	0	0	8	14	19	31	72
Rumen fill score	0	0	7	8	14	24	53
Both combined *	0	0	13	18	28	42	101

^+^ It was assumed that herbage on pasture was sufficient on days 1 and 2, because meadow hay was fed ad libitum on day 1 before grazing began and compressed sward height was at its highest during these days (Figure 1). * Classified as “scarce” as soon as either the milk yield or rumen fill reference indicator classified a cow-day as “scarce”.

**Table 5:** Number of cow-days with scarce herbage availability identified by generalized linear model (GLM) and random forest model (RFM) on days 1 to 6 across six grazing cycles and 20 cows. The number of total cow-day observations per grazing day is written in brackets.

Prediction Approach	Day 1 (*n* = 91)	Day 2 (*n* = 92)	Day 3 (*n* = 101)	Day 4 (*n* = 101)	Day 5 (*n* = 99)	Day 6 (*n* = 99)	Total (*n* = 583)
GLM	0	4	7	12	13	21	57
RFM	0	2	9	10	8	16	45

**Table 6:** Evaluation metrics for the binary classification via generalized linear model (GLM) or random forest model (RFM) as compared to the reference classifications based on milk yield (MY_Ref_), rumen fill scoring (RF_Ref_), and a combination of both (COMB_Ref_) across all observations (*n* = 583).

Predicted Versus Reference Classification	Sensitivity	Specificity	PPV	AUC
GLM versus MY_Ref_	0.10 (0.19)	0.90 (0.84)	0.12 (0.15)	0.50 (0.52)
RFM versus MY_Ref_	0.06 (0.25)	0.92 (0.81)	0.09 (0.16)	0.49 (0.53)
GLM versus RF_Ref_	0.15 (0.25)	0.91 (0.84)	0.14 (0.14)	0.53 (0.54)
RFM versus RF_Ref_	0.06 (0.28)	0.92 (0.82)	0.07 (0.13)	0.49 (0.55)
GLM versus COMB_Ref_	0.12 (0.22)	0.91 (0.85)	0.21 (0.23)	0.51 (0.53)
RFM versus COMB_Ref_	0.06 (0.24)	0.92 (0.82)	0.13 (0.21)	0.49 (0.53)

Predictions were based either on all eight behavioral variables presented in Table 2 or on only five variables, where the variables “rumination chews per bolus”, “head movement activity”, and “lying bouts” were excluded, because they were identified as predictors of low relevance (see Section 3.3). Results of the prediction with only five variables are provided in parentheses. PPV: positive predictive value, AUC: area under receiver operating characteristic curve.

## Data Availability

The data presented in this study are available in Supplementary Materials (test dataset) and in Shafiullah et al. [19] (training dataset).

## Supplementary Materials

The following are available online at https://www.mdpi.com/article/10.3390/s22030968/s1, File S1: Dataset_final589.txt.

## Appendix A

**Table A1:** Calibrations (ordinary linear regressions) used to convert the compressed sward height measured with a rising plate meter (x) into a herbage mass estimate (y).

Ordinary Linear Regression	Experimental Month	Sample Number	R^2^
y = −1.8173 + 18.012 × x	May	9	0.70
y = 184.8 + 17.794 × x	July	15	0.83
y = 1368 + 15.069 × x	September	15	0.34

**Table A2:** Evaluation metrics for the binary classification with a generalized linear model (GLM) and a random forest model (RFM) approach in different subgroups of cows. The reference was a combination of milk yield and rumen fill reference indicators.

Subgroup (Number of Cow-Days)	Prediction Approach	Sensitivity	Specificity	PPV	AUC
Primiparous cows (*n* = 209)	GLM	0.06 (0.09)	0.91 (0.86)	0.11 (0.11)	0.48 (0.47)
	RFM	0.06 (0.37)	0.92 (0.76)	0.13 (0.24)	0.49 (0.57)
Multiparous cows (*n* = 374)	GLM	0.15 (0.29)	0.91 (0.84)	0.26 (0.28)	0.53 (0.56)
	RFM	0.03 (0.17)	0.92 (0.84)	0.08 (0.19)	0.48 (0.51)
High-performing cows (*n* = 186)	GLM	0.22 (0.30)	0.92 (0.82)	0.28 (0.19)	0.57 (0.56)
	RFM	0.04 (0.17)	0.93 (0.83)	0.08 (0.13)	0.48 (0.50)
Medium-performing cows (*n* = 212)	GLM	0.15 (0.29)	0.89 (0.87)	0.20 (0.30)	0.52 (0.58)
	RFM	0.06 (0.26)	0.90 (0.82)	0.10 (0.22)	0.48 (0.54)
Low-performing cows (*n* = 185)	GLM	0.05 (0.11)	0.91 (0.85)	0.14 (0.19)	0.48 (0.48)
	RFM	0.02 (0.25)	0.94 (0.79)	0.11 (0.27)	0.48 (0.52)
Cows with grazing experience (*n* = 274)	GLM	0.20 (0.30)	0.91 (0.83)	0.30 (0.25)	0.56 (0.56)
	RFM	0.05 (0.27)	0.93 (0.83)	0.11 (0.23)	0.49 (0.55)
Cows without grazing experience (*n* = 309)	GLM	0.05 (0.16)	0.90 (0.87)	0.11 (0.21)	0.48 (0.51)
	RFM	0.04 (0.21)	0.91 (0.80)	0.08 (0.19)	0.47 (0.51)
High BCS (*n* = 219)	GLM	0.03 (0.10)	0.91 (0.86)	0.06 (0.13)	0.47 (0.48)
	RFM	0.00 (0.23)	0.92 (0.79)	0.00 (0.20)	0.46 (0.51)
Medium BCS (*n* = 223)	GLM	0.15 (0.23)	0.88 (0.81)	0.25 (0.25)	0.51 (0.52)
	RFM	0.09 (0.21)	0.93 (0.83)	0.24 (0.25)	0.51 (0.52)
Low BCS (*n* = 141)	GLM	0.27 (0.47)	0.94 (0.88)	0.33 (0.32)	0.60 (0.67)
	RFM	0.00 (0.33)	0.92 (0.82)	0.00 (0.18)	0.46 (0.58)
Empty cows (*n* = 220)	GLM	0.04 (0.08)	0.91 (0.81)	0.05 (0.05)	0.47 (0.44)
	RFM	0.04 (0.32)	0.90 (0.79)	0.05 (0.17)	0.47 (0.56)
Cows in gestation (*n* = 363)	GLM	0.14 (0.26)	0.91 (0.87)	0.29 (0.36)	0.53 (0.57)
	RFM	0.04 (0.21)	0.94 (0.83)	0.14 (0.24)	0.49 (0.52)

Predictions were based either on all eight behavioral variables presented in Table 2 or on only five variables, where the variables “rumination chews per bolus”, “head movement activity”, and “lying bouts” were excluded, because they were identified as predictors of low relevance in Section 3.3. Results of the latter are provided in parentheses. BCS: body condition score, PPV: positive predictive value, AUC: area under receiver operating characteristic curve.

**Table A3:** Results of the confusion matrixes for the prediction * via generalized linear model (GLM) and random forest model (RFM °) in comparison to the reference indicators of milk yield and rumen fill scoring as well as a combination of both (*n* = 583).

Reference Indicator	Prediction Approach	True Negative	False Negative	True Positive	False Positive
Milk yield	GLM	461	65	7	50
	RFM	471	68	4	40
Rumen fill scoring	GLM	481	45	8	49
	RFM	490	49	4	40
Both combined	GLM	437	89	12	45
	RFM	444	95	6	38

* including all eight behavioral variables as shown in Table 2. ° data may differ from those in Table 5, because rerunning the RFM prediction produced slightly different results.

## References

[1] Neethirajan S., Kemp B. (2021). Digital Livestock Farming. Sens. Bio-Sens. Res..

[2] Akhigbe B.I., Munir K., Akinade O., Akanbi L., Oyedele L.O. (2021). IoT Technologies for Livestock Management: A Review of Present Status, Opportunities, and Future Trends. Big Data Cogn. Comput..

[3] Slob N., Catal C., Kassahun A. (2021). Application of machine learning to improve dairy farm management: A systematic literature review. Prev. Vet. Med..

[4] Benos L., Tagarakis A.C., Dolias G., Berruto R., Kateris D., Bochtis D. (2021). Machine Learning in Agriculture: A Comprehensive Updated Review. Sensors.

[5] Cockburn M. (2020). Review: Application and Prospective Discussion of Machine Learning for the Management of Dairy Farms. Animals.

[6] Stygar A.H., Gómez Y., Berteselli G.V., Dalla Costa E., Canali E., Niemi J.K., Llonch P., Pastell M. (2021). A Systematic Review on Commercially Available and Validated Sensor Technologies for Welfare Assessment of Dairy Cattle. Front. Vet. Sci..

[7] Shalloo L., O’ Donovan M., Leso L., Werner J., Ruelle E., Geoghegan A., Delaby L., O’Leary N. (2018). Review: Grass-based dairy systems, data and precision technologies. Animal.

[8] Hamidi D., Komainda M., Tonn B., Harbers J., Grinnell N.A., Isselstein J. (2021). The Effect of Grazing Intensity and Sward Heterogeneity on the Movement Behavior of Suckler Cows on Semi-natural Grassland. Front. Vet. Sci..

[9] Murphy D.J., O’Brien B., Murphy M.D. (2020). Development of a grass measurement optimisation tool to efficiently measure herbage mass on grazed pastures. Comput. Electron. Agric..

[10] Deming J., Gleeson D., O’Dwyer T., Kinsella J., O’Brien B. (2018). Measuring labor input on pasture-based dairy farms using a smartphone. J. Dairy Sci..

[11] Murphy D.J., O’ Brien B., Hennessy D., Hurley M., Murphy M.D. (2021). Evaluation of the precision of the rising plate meter for measuring compressed sward height on heterogeneous grassland swards. Precis. Agric..

[12] Sishodia R.P., Ray R.L., Singh S.K. (2020). Applications of Remote Sensing in Precision Agriculture: A Review. Remote Sens..

[13] Sinde-González I., Gil-Docampo M., Arza-García M., Grefa-Sánchez J., Yánez-Simba D., Pérez-Guerrero P., Abril-Porras V. (2021). Biomass estimation of pasture plots with multitemporal UAV-based photogrammetric surveys. Int. J. Appl. Earth Obs. Geoinf..

[14] Nickmilder C., Tedde A., Dufrasne I., Lessire F., Tychon B., Curnel Y., Bindelle J., Soyeurt H. (2021). Development of Machine Learning Models to Predict Compressed Sward Height in Walloon Pastures Based on Sentinel-1, Sentinel-2 and Meteorological Data Using Multiple Data Transformations. Remote Sens..

[15] Decruyenaere V., Buldgen A., Stilmant D. (2009). Factors affecting intake by grazing ruminants and related quantification methods: A review. BASE.

[16] O’Driscoll K., Lewis E., Kennedy E. (2019). Effect of feed allowance at pasture on the lying behaviour of dairy cows. Appl. Anim. Behav. Sci..

[17] Werner J., Umstatter C., Kennedy E., Grant J., Leso L., Geoghegan A., Shalloo L., Schick M., O’Brien B. (2019). Identification of possible cow grazing behaviour indicators for restricted grass availability in a pasture-based spring calving dairy system. Livest. Sci..

[18] Rombach M., Südekum K.H., Münger A., Schori F. (2019). Herbage dry matter intake estimation of grazing dairy cows based on animal, behavioral, environmental, and feed variables. J. Dairy Sci..

[19] Shafiullah A.Z., Werner J., Kennedy E., Leso L., O’Brien B., Umstätter C. (2019). Machine Learning Based Prediction of Insufficient Herbage Allowance with Automated Feeding Behaviour and Activity Data. Sensors.

[20] Post C., Rietz C., Büscher W., Müller U. (2021). The Importance of Low Daily Risk for the Prediction of Treatment Events of Individual Dairy Cows with Sensor Systems. Sensors.

[21] Spengler Neff A., Notz C., Ivemeyer S., Walkenhorst M., FiBL (2015). Koerper-Konditions-Beurteilung.

[22] Pérez-Ramírez E., Peyraud J., Delagarde R. (2009). Restricting daily time at pasture at low and high pasture allowance: Effects on pasture intake and behavioral adaptation of lactating dairy cows. J. Dairy Sci..

[23] Gulati A., Galvin N., Kennedy E., Lewis E., McManus J.J., Fenelon M.A., Guinee T.P. (2019). Effect of reducing daily herbage allowance during early lactation on composition and processing characteristics of milk from spring-calved herds. Int. Dairy J..

[24] Dessauge F., Lollivier V., Ponchon B., Bruckmaier R., Finot L., Wiart S., Cutullic E., Disenhaus C., Barbey S., Boutinaud M. (2011). Effects of nutrient restriction on mammary cell turnover and mammary gland remodeling in lactating dairy cows. J. Dairy Sci..

[25] Gross J., van Dorland H.A., Bruckmaier R.M., Schwarz F.J. (2011). Performance and metabolic profile of dairy cows during a lactational and deliberately induced negative energy balance with subsequent realimentation. J. Dairy Sci..

[26] Mosimann E., Stettler M., AGFF (2004). Weiden von Milchkühen: Berechnung der angepassten Besatzstärke. Weide- und Alpwirtschaft Information W10.

[27] Zehner N., Umstätter C., Niederhauser J.J., Schick M. (2017). System specification and validation of a noseband pressure sensor for measurement of ruminating and eating behavior in stable-fed cows. Comput. Electron. Agric..

[28] Werner J., Leso L., Umstatter C., Niederhauser J., Kennedy E., Geoghegan A., Shalloo L., Schick M., O’Brien B. (2018). Evaluation of the RumiWatchSystem for measuring grazing behaviour of cows. J. Neurosci. Methods.

[29] Reith S., Hoy S. (2012). Relationship between daily rumination time and estrus of dairy cows. J. Dairy Sci..

[30] Zaaijer D., Noordhuizen J.P.T.M. (2003). A novel scoring system for monitoring the relationship between nutritional efficiency and fertility in dairy cows. Ir. Vet. J..

[31] Burfeind O., Sepúlveda P., von Keyserlingk M.A.G., Weary D.M., Veira D.M., Heuwieser W. (2010). Technical note: Evaluation of a scoring system for rumen fill in dairy cows. J. Dairy Sci..

[32] Agroscope Frisst die Kuh Genug?. https://www.agroscope.admin.ch/agroscope/de/home/aktuell/newsroom/2021/04-07_bewertung-hungergrube-kuh.html.

[33] Schneider M., Hart L., Gallmann E., Umstaetter C. (2022). A Novel Chart to Score Rumen Fill Following Simple Sequential Instructions.

[34] Huhtanen P., Hetta M. (2012). Comparison of feed intake and milk production responses in continuous and change-over design dairy cow experiments. Livest. Sci..

[35] Faverdin P., Baratte C., Delagarde R., Peyraud J.L. (2011). GrazeIn: A model of herbage intake and milk production for grazing dairy cows. 1. Prediction of intake capacity, voluntary intake and milk production during lactation. Grass Forage Sci..

[36] Hristov A.N., Price W.J., Shafii B. (2004). A Meta-Analysis Examining the Relationship Among Dietary Factors, Dry Matter Intake, and Milk and Milk Protein Yield in Dairy Cows. J. Dairy Sci..

[37] Delagarde R., Faverdin P., Baratte C., Peyraud J.L. (2011). GrazeIn: A model of herbage intake and milk production for grazing dairy cows. 2. Prediction of intake under rotational and continuously stocked grazing management. Grass Forage Sci..

[38] Oudshoorn F.W., Cornou C., Hellwing A.L.F., Hansen H.H., Munksgaard L., Lund P., Kristensen T. (2013). Estimation of grass intake on pasture for dairy cows using tightly and loosely mounted di- and tri-axial accelerometers combined with bite count. Comput. Electron. Agric..

[39] Fisher R. (1925). Statistical Methods for Research Workers.

[40] R Core Team R: A Language and Environment for Statistical Computing. http://www.R-project.org/.

[41] Liaw A., Wiener M. (2002). Classification and Regression by randomForest. R News.

[42] Kuhn M. (2008). Building predictive models in R using the caret package. J. Stat. Softw..

[43] Lindstrom M.J., Bates D.M. (1990). Nonlinear Mixed Effects Models for Repeated Measures Data. Biometrics.

[44] Hastie T.J., Chambers J.M., Hastie T.J. (1992). Generalized additive models. Statistical Models in S.

[45] Wickham H. (2016). ggplot2: Elegant Graphics for Data Analysis.

[46] Herve L., Quesnel H., Veron M., Portanguen J., Gross J.J., Bruckmaier R.M., Boutinaud M. (2019). Milk yield loss in response to feed restriction is associated with mammary epithelial cell exfoliation in dairy cows. J. Dairy Sci..

[47] Götze K., Crivellaro P., Pieper L., Engelhard T., Staufenbiel R. (2019). Assessment of rumen fill in dairy cows for evaluation of the individual feed intake in herd management. Tierartzl. Prax. Ausg. G Grosstiere Nutztiere.

[48] Bareille N., Beaudeau F., Billon S., Robert A., Faverdin P. (2003). Effects of health disorders on feed intake and milk production in dairy cows. Livest. Prod. Sci..

[49] Norring M., Häggman J., Simojoki H., Tamminen P., Winckler C., Pastell M. (2014). Short communication: Lameness impairs feeding behavior of dairy cows. J. Dairy Sci..

[50] Baumgard L.H., Collier R.J., Bauman D.E. (2017). A 100-Year Review: Regulation of nutrient partitioning to support lactation. J. Dairy Sci..

[51] Post C., Rietz C., Büscher W., Müller U. (2020). Using Sensor Data to Detect Lameness and Mastitis Treatment Events in Dairy Cows: A Comparison of Classification Models. Sensors.

[52] Schils R., Philipsen B., Hoekstra N., Holshof G., Zom R., Hoving I., van Reenen K., Stienezen M., Klootwijk C., van der Werf J. (2019). Amazing Grazing: A Public and Private Partnership to Stimulate Grazing Practices in Intensive Dairy Systems. Sustainability.

[53] Halachmi I., Ben Meir Y., Miron J., Maltz E. (2016). Feeding behavior improves prediction of dairy cow voluntary feed intake but cannot serve as the sole indicator. Animal.

[54] Andriamandroso A.L.H., Lebeau F., Bindelle J. Changes in biting characteristics recorded using the inertial measurement unit of a smartphone reflect differences in sward attributes. Proceedings of the 7th European Conference on Precision Livestock Farming.

[55] Gregorini P., DelaRue B., McLeod K., Clark C.E.F., Glassey C.B., Jago J. (2012). Rumination behavior of grazing dairy cows in response to restricted time at pasture. Livest. Sci..

[56] Kennedy E., McEvoy M., Murphy J.P., O’Donovan M. (2009). Effect of restricted access time to pasture on dairy cow milk production, grazing behavior, and dry matter intake. J. Dairy Sci..

[57] Pérez-Ramírez E., Delagarde R., Delaby L. (2008). Herbage intake and behavioural adaptation of grazing dairy cows by restricting time at pasture under two feeding regimes. Animal.

[58] Paulenz E., Gygax L., Barth K., Hart L., Hillmann E. (2022). Effect of Sward Height on the Behavior of Dairy Cows in a Rotational Grazing System.

[59] Krizsan S.J., Sairanen A., Höjer A., Huhtanen P. (2014). Evaluation of different feed intake models for dairy cows. J. Dairy Sci..

[60] Codrea M.C., Højsgaard S., Friggens N.C. (2011). Differential smoothing of time-series measurements to identify disturbances in performance and quantify animal response characteristics: An example using milk yield profiles in dairy cows1. J. Anim. Sci..

